# Evaluation of Xylooligosaccharides Production for a Specific Degree of Polymerization by Liquid Hot Water Treatment of Tropical Hardwood

**DOI:** 10.3390/foods10020463

**Published:** 2021-02-20

**Authors:** Soo-Kyeong Jang, Jong-Hwa Kim, June-Ho Choi, Seong-Min Cho, Jong-Chan Kim, Hoyong Kim, In-Gyu Choi

**Affiliations:** 1 Department of Wood Science, Faculty of Forestry, The University of British Columbia, 2424 Main Mall, Vancouver, BC V6T 1Z4, Canada; sookyeong.jang@ubc.ca; 2 Department of Forest Science, College of Agriculture and Life Science, Seoul National University, Seoul 08826, Korea; wmfty@snu.ac.kr (J.-H.K.); jhchoi1990@snu.ac.kr (J.-H.C.); csmin93@snu.ac.kr (S.-M.C.); whdcnpdls@snu.ac.kr (J.-C.K.); 3 Center for Bio-Based Chemistry, Korea Research Institute of Chemical Technology, Ulsan 44429, Korea; 4 Research Institute of Agriculture and Life Sciences, College of Agriculture and Life Sciences, Seoul National University, Seoul 08826, Korea

**Keywords:** xylooligosaccharides (XOS), liquid hot water treatment, *Eucalyptus pellita*, combined severity factor, XOS production score

## Abstract

*Eucalyptus pellita* is known as attractive biomass, and it has been utilized for eucalyptus oil, furniture, and pulp and paper production that causes a significant amount of byproducts. Liquid hot water treatment depending on combined severity factor (CSF) was subjected to isolate hemicellulose fraction from *E. pellita* and to produce xylooligosaccharides (XOS). The xylan extraction ratio based on the initial xylan content of the feedstock was maximized up to 77.6% at 170 °C for 50 min condition (CSF: 1.0), which had accounted for XOS purity of 76.5% based on the total sugar content of the liquid hydrolysate. In this condition, the sum of xylobiose, xylotriose, and xylotetraose which has a low degree of polymerization (DP) of 2 to 4 was determined as 80.6% of the total XOS. The highest XOS production score established using parameters including the xylan extraction ratio, XOS purity, and low DP XOS ratio was 5.7 at CSF 1.0 condition. XOS production score evaluated using the CSF is expected to be used as a productivity indicator of XOS in the industry (R-squared value: 0.92).

## 1. Introduction

Xylooligosaccharides (XOS) has been recognized as one of the most notable foods and food additives because it has beneficial influences on the human body that reported in the previous studies [1]. It contributes primarily to the proliferation of bifidobacteria that makes good intestine condition and inhibit the pathogenic and putrefactive microorganisms [2]. XOS can be supplied as low-calorie foods for weight loss and has many advantageous effects such as reduction of cardiovascular symptoms, alleviation of dental decaying, activation of calcium absorption, and improvement of bowel function [3]. Besides XOS has a high price than that of xylose even though both products are commonly originated xylan [4]. Therefore, the value of lignocellulosic biomass can be advanced as an XOS source because lignocellulosic biomass contains a large amount of xylan and it is abundant worldwide.

Generally, conventional production of XOS follows some of the process: xylan extraction from biomass, enzymatic hydrolysis using xylanase, and purification [5]. However, the high cost and long hydrolysis time of xylanase utilization have been recognized as major drawbacks of the conventional process for XOS production [6]. To overcome these limitations, the hydrothermal methods as non-biological approaches have been considered for producing XOS [7]. However, hemicellulose structure can be easily decomposed into monomeric sugars by thermal energy and acidic condition [8]. Therefore, appropriate conditions should be investigated for suitable XOS production [9]. 

Eucalyptus species are native to Australia but are now widely inhabited in subtropical and tropical regions [10]. For instance, *Eucalyptus globulus* or *Eucalyptus grandis* has been employed as a feedstock in many studies about pretreatment or enzymatic hydrolysis due to extensive afforestation in the USA at the 1900s [11]. Meanwhile, *Eucalyptus pellita* has been few applied to pretreatment researches because it has recently been widely used for artificial planting in South or Southeast Asia [10]. *E. pellita* has many advantages for afforestation such as the fast growth rate, high environmental robustness, and good resistance to diseases [12]. However, the high density (~990 g/m^3^) and high lignin content (~35%) of *E. pellita* were considered drawbacks as it may require more energy and chemicals to degrade the cell-wall structure [13]. *E. pellita* has been utilized in the production of eucalyptus oil, furniture, and pulp and paper that causes a significant amount of byproducts such as twigs, branches, and sawdust [14].

In this study, liquid hot water (LHW) treatment, which is one of the hydrothermal treatments without any catalyst, was performed using *E. pellita* for XOS production. A trend of xylan decomposition from *E. pellita* was investigated depending on the conditions of LHW treatment with various combined severity factors (CSF), and XOS content was evaluated as the degree of polymerization. Additionally, we suggested the parameters which are related to the XOS production cost and determined the XOS production score based on the parameters. In addition, the relationship between XOS production score and CSF value was evaluated. The solid residues of LHW treatment were employed enzymatic hydrolysis for evaluating the cell-wall degradation.

## 2. Materials and Methods

### 2.1. Feedstock

*Eucalyptus pellita* was used for artificial reforestation in Indonesia, and its powder was generously supplied by the Korea Research Institute of Chemical Technology (Daejeon, Republic of Korea). Then, *E. pellita* powder was treated by twin-extruder with distilled water to keep the particle size constant as 0.5 mm. The milled *E. pellita*, the feedstock of this study, has approximately 59.3% of moisture content, and stored in refrigerator at 4 °C for employing next processes. 

### 2.2. Liquid Hot Water Treatment

Liquid hot water (LHW) treatment was conducted using the milled *E. pellita* for producing xylooligosaccharides (XOS). A total of 122.9 g of wet feedstock (equaled to 50 g of oven-dried weight) and 500 mL of distilled water were loaded into a 1000-mL capable batch type reactor (HR-8300, Hanwoul Engineering Inc., Gunpo, Republic of Korea) (solid:liquid ratio = 1:10 (*w*/*v*)). This stainless steel (SUS 316) reactor was equipped a metal jacket that heated until the target temperature (170, 180, 190, or 210 °C) during the designated heat-up time (50 min). The target temperature (reaction temperature) maintained for 10, 20, 30, 50, or 70 min. After the LHW treatment, the reactor was cooled to 50 °C using a quenching chamber. Then, liquid hydrolysate was separated from solid fraction by filter paper (No. 52, Hyundai micro CO., Seoul, Korea). For determination of monomeric sugars, sugar derivatives, and XOS contents, the liquid hydrolysate was filtered using a 0.45 μm membrane filter (Advantec, Tokyo, Japan). On the other hand, the solid fraction was washed with distilled water, and kept at 4 °C for enzymatic hydrolysis. 

The reaction temperature (T(t), °C), time (t, min), and pH value of each LHW treatment conditions were subjected to calculate the combined severity factor (CSF) using the below formula [15]:

(1) $$\mathrm{CSF}\;=\;log^{\left(t\;\times \;e^{\left(\frac{T\left(t\right)-100}{14.75}\right)}\right)}\;-pH$$

### 2.3. Chemical Composition of Solid Fractions

Water-insoluble solid (WIS) recovery rate of solid residue after LHW treatment was calculated by weighing of the wet solid residue and estimating its moisture content. Meanwhile, amount of acid-insoluble lignin (AIL) and acid-soluble lignin (ASL) from feedstock and solid fractions obtained by LHW treatment were determined according to Laboratory Analytical Procedure of the National Renewable Energy Laboratory (NREL) [16]. 0.3 g of oven-dried samples were soaked in 3 mL of 72% sulfuric acid at 30 °C for 1 h. Then, 84 mL of distilled water was added to reach 4% sulfuric acid solution. The mixture was reacted by autoclave at 121 °C for 1 h. After the reaction, the residues were recovered by a glass filter and weighed for determining AIL. Meanwhile, ASL was determined by measuring the absorbance of the diluted filtrate at 205 nm wavelength using a UV-visible spectrophotometer (UV-1601 PC, Shimadzu, Japan). 

Monomeric sugar (glucose, xylose, mannose, galactose, and arabinose) content in the feedstock and solid fractions after LHW treatment was determined using ion chromatography (ICS2500, Thermo Dionex, Palo Alto, CA, USA). It equipped with a CarboPac PA-1 column (250 × 4 mm, Dionex, Palo Alto, CA, USA) and a pulsed amperometric detector (HP 1100, Hewlett Packard, Palo Alto, CA, USA). The analysis was conducted at 40 °C using potassium hydroxide as an eluent at a flow rate of 1 mL/min, and the injection volume of sample was 10 μL. The detailed methods are referred by previous study [17].

### 2.4. Chemical Composition of Liquid Hydrolysates

XOS (xylobiose, xylotriose, xylotetraose, xylopentaose, and xylohexaose) content in the liquid hydrolysate after LHW treatment was determined using ion chromatography (ICS5000, Thermo Dionex, Palo Alto, CA, USA). It equipped with a CarboPac SA-10 column (250 × 4 mm, Dionex, Palo Alto, CA, USA) and same detector, which is used for determination of the monomeric sugar. The xylose and XOS analysis was conducted at 32 °C using 100 mM and 200 mM of sodium hydroxide with 100 mM and 1 mM of sodium acetate at a flow rate of 0.85 mL/min. In addition, the injection volume of sample was 10 μL. The XOS standard solutions, xylobiose, xylotriose, xylotetraose, xylopentaose, and xylohexaose, were purchased by Megazyme (Wicklow, Ireland) for making calibration curves, and it used for quantitative analysis.

The amount of sugar derivatives (furfural, 5-hydroxymethylfurfural (5-HMF), levulinic acid, acetic acid, and formic acid) in the liquid hydrolysate after LHW treatment were determined using a high performance liquid chromatography (Ultimate 3000, Thermo Dionex, Palo Alto, CA, USA) with a Aminex 87H column. The sugar derivatives analysis was conducted at 40 °C using 0.1 N of sulfuric acid as an eluent at a flow rate of 0.5 mL/min with 10 μL of injection volume of sample. The standard solutions of furfural, 5-HMF, levulinic acid, acetic acid, and formic acid was purchased by Sigma-Aldrich Korea CO. (Yongin, Korea) for making calibration curves. 

Conversion ratio of xylose or XOS, Xylan extraction ratio, XOS purity, low DP XOS ratio, XOS production factor were calculated as following formula:

(2) $$\mathrm{Conversion}\;\mathrm{ratio}\;(\%)\;=\;\frac{Xylose\;or\;XOS\;content\;in\;liquid\;hydrolysate\;\left(g\right)}{Xylan\;content\;in\;initial\;biomass\;\left(g\right)}\;\times 100$$

(3) $$\mathrm{Xylan}\;\mathrm{extraction}\;\mathrm{ratio}\;(\%)\;=\;\frac{Xylose\;and\;XOS\;content\;in\;liquid\;hydrolysate\;\left(g\right)}{Xylan\;content\;in\;initial\;biomass\;\left(g\right)}\;\times 100$$

(4) $$\mathrm{XOS}\;\mathrm{purity}\;(\%)\;=\;\frac{Total\;XOS\;content\;\left(DP\;2-6\right)\;in\;liquid\;hydrolysate\;\left(g\right)}{Total\;sugar\;content\;in\;liquid\;hydrolysate\;\left(g\right)}\;\times 100$$

(5) $$\mathrm{Low}\;\mathrm{DP}\;\mathrm{XOS}\;\mathrm{ratio}\;(\%)\;=\;\frac{Low\;DP\;XOS\;content\;\left(DP\;2-4\right)\;in\;liquid\;hydrolysate\;\left(g\right)}{Total\;XOS\;content\;\left(DP\;2-6\right)\;in\;liquid\;hydrolysate\;\left(g\right)}\times 100$$

(6) $$\mathrm{XOS}\;\mathrm{production}\;\mathrm{score}\;=\;log^{\left(Xylan\;extraction\;ratio\;\times \;XOS\;purity\;\times \;Low\;DP\;XOS\;ratio\right)}\times 100$$

### 2.5. Enzymatic Hydrolysis

After LHW treatments, the feedstock and solid fractions (equivalent to the 1 g oven-dried weight) were subjected to enzymatic hydrolysis. These substrates were swelled with 50 mM sodium acetate buffer (pH 5.0). In addition, a commercial cellulase cocktail, Cellic CTec2 (Novozymes, Bagsværd, Denmark), was used as 15 FPU/g substrate, and it mixed with the substrates and buffer. The substrate, buffer, and enzyme solution were incubated at 50 °C for 72 h in a hybridization incubator (combi-D24, FINEPCR, Gunpo, Korea). Then, the mixture was filtered by filter paper, and the residues were dried in an oven (HB-501M, Hanbaek Scientific Technology, Bucheon, Republic of Korea) at 65 °C for 24 h. Enzymatic digestibility was calculated by oven-dried weight as follows;

Enzymatic digestibility (%) = (weight of substrate (g, equal to 1) − weight of residues on the filter paper (g)) × 100(7)

On the other hand, glucose yield was determined as follows;

(8) $$\mathrm{Glucose}\;\mathrm{yield}\;(\%)\;=\;\frac{Glucose\;content\;in\;filtrate\;\left(g\right)}{Glucan\;content\;in\;initial\;biomass\;\left(g\right)}\;\times 100$$

### 2.6. Statistical Analysis

All of the experimental results were presented as mean ± standard deviation. In addition, the mean values were conducted by Student’s t-test using spreadsheet software (Excel) with 0.95 confidence level. All experiments were triplicated.

## 3. Results and Discussion

### 3.1. Physicochemical Characteristics of Solid Fractions with Mass Balance Analysis

A change of water insoluble solid (WIS) recovery rate from *E. pellita* after LHW treatment is shown in Table 1. WIS recovery rate decreased to 76.6% at 210 °C for 10 min which was the highest reaction temperature among conditions of LHW treatment. WIS recovery rate also decreased by extension of the reaction time among similar reaction temperature conditions, for example, 90.6% of WIS recovery rate at 170 °C for 10 min was dropped to 80.8% at 170 °C for 70 min. The severity of hydrothermal treatment is usually determined by the supplied thermal energy which is primarily controlled by reaction temperature or time. Therefore, a harsh condition such as 210 °C for 10 min in this study gave significant damage to the chemical structure of *E. pellita*. The severity of hydrothermal treatment is also influenced by the amount of catalyst input. Although no catalyst was employed for LHW treatment, acetic acid can be released into a solvent that is originated from the lignocellulosic biomass [18]. In the case of hardwood, hydroxyl groups are substituted with acetyl group at C2 and/or C3 carbon of the xylose unit in the hemicellulose chain [19]. In addition, the acetyl group is more released as an increase of the severity of hydrothermal treatment that enhances the acidity of the solvent [20]. Consequently, a low WIS recovery rate of solid residues at high reaction temperature and long reaction time might be caused by an increase of acetyl group decomposition as well as thermal energy.

Table 2 summarizes the structural sugars (glucan, xylan, galactan, mannan, and arabinan) content of *E. pellita* and the solid residues after LHW treatment. Generally, the hemicellulose in hardwood is primarily composed of glucuronoxylan (15–30%, % of wood), while that of softwood mainly consists of galactoglucomannan (10–15%, % of wood) [21]. This difference about a kind of the dominant backbone in hemicellulose structure is primarily determined by the amount of xylan or mannan in biomass. Thus, *E. pellita* might be had a similar hemicellulose structure with hardwood because of the high amount of xylan.

Glucan (50.9%) occupied the largest portion of structural sugars in *E. pellita*, and glucan content was roughly constant even though changes of reaction temperature and time during LHW treatment. Cellulose has different properties compared to hemicellulose such as only composed of glucose, linear polymer topology, no substitution at side group, the existence of crystalline region, low reactivity, and poor solubility to water [22]. These features of cellulose provide resistance that is not easily decomposed by hydrothermal treatment. Meanwhile, the amount of glucan in the solid residues after LHW treatment was decreased slightly than that in *E. pellita*. It is assumed that the small amount of glucose, which was dissolved into liquid hydrolysate, was originated from side chains of hemicellulose, not cellulose.

As the reaction temperature increased at 180 °C for 10 min, xylan content was dramatically decreased to 3.4%, and little amount of xylan (0.5%) remained in the solid residue at 210 °C for 10 min. This decomposition trend was quite different from the case of cellulose mentioned above, and it denoted that hemicellulose has more reactive and susceptible under the condition of hydrothermal reaction [23]. On the other hand, mannan and arabinan were fully decomposed at 170 °C, and galactan dissipated above 170 °C conditions in the solid residue.

### 3.2. Chemical Composition of Liquid Hydrolysates after LHW Treatment

**Figure 1:**
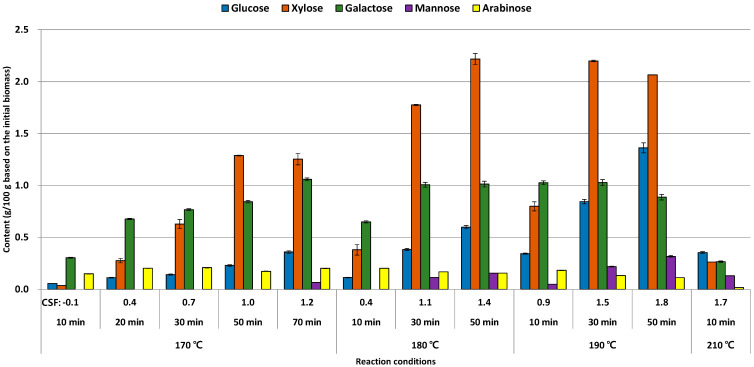
Monomeric sugar content (g/100 g initial biomass) of liquid hydrolysate after LHW treatment from *E. pellita*.

The amount of glucose was ranged from 0.1% (170 °C for 10 min) to 1.2% (190 °C for 50 min) (Figure 1). Considering the glucan content in *E. pellita* (50.9%), a very small amount of glucose was released into the liquid hydrolysate. In the same reaction temperature condition at 170 °C or 180 °C or 190 °C, glucose content was increased as the reaction time was prolonged. As a result, the decomposition of the glucan might be enhanced by affording enough thermal energy. 

Xylose was more released than glucose into the liquid hydrolysate, and the maximum content was 2.2% under conditions at 180 °C for 50 min and 190 °C for 30 min. The trend of glucose content which was increased as an extension of reaction time in the same reaction temperature was also denoted in the case of xylose. However, xylose content was decreased slightly (2.1%) under high reaction temperature and time like 190 °C for 50 min. It is known that the monomeric sugars derived from hemicellulose could be easily converted into various sugar derivatives during hydrothermal treatment [24]. In addition, this conversion reaction might be enhanced by an increase in reaction temperature, reaction time, and concentration of acid catalysts [7]. This point could be observed at 210 °C for 10 min condition, and all monomeric sugars were diminished dramatically less than 0.3% in this condition.

**Figure 2:**
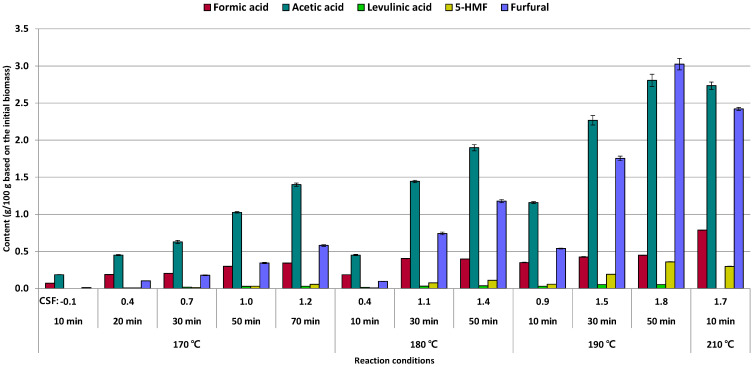
Sugar derivatives content (g/100 g initial biomass) of liquid hydrolysate after LHW treatment from *E. pellita*.

The content of sugar derivatives (formic acid, acetic acid, levulinic acid, 5-hydroxymethylfurfural (5-HMF), and furfural) are shown in Figure 2. Acetic acid has been pointed out as a major factor for allowing acid hydrolysis during LHW treatment due to enhancing the acidity. Among the sugar derivatives, acetic acid was largely dissolved under most conditions of LHW treatment. The amount of acetic acid in the liquid hydrolysate was increased steadily as an extension of reaction time with the same reaction temperature (170 °C or 180 °C or 190 °C) or an increase of reaction temperature with the same reaction time (10 min or 30 min or 50 min). Both of the condition changes allowed to increase the thermal energy to biomass. The maximum content of acetic acid (2.8%) was observed at 190 °C for 50 min, but it is similar to acetic acid content at 210 °C for 10 min (2.7%).

The amount of furfural was less than 1% at 170 °C of reaction temperature, but it was increased sharply up to 3.0% at 190 °C for 50 min. Furfural was converted typically from pentose through isomerization, enolization, and dehydration during acid hydrolysis [25]. Thus, a sufficient amount of xylose or arabinose in the liquid hydrolysate could be easily converted to furfural during the LHW treatment. In addition to a significant amount of acetic acid was released simultaneously under the severe reaction condition (190 °C for 50 min) that might accelerate furfural conversion from pentose. However, the furfural content was slightly decreased at 210 °C for 10 min because of further conversion reaction from furfural to formic acid or humin formation [26].

Few amounts of 5-HMF and levulinic acid were observed in the liquid hydrolysates under all conditions of LHW treatment. These sugar derivatives have known as mainly converted from hexose [27]. However, glucose, galactose, and mannose as a source of 5-HMF or levulinic acid were released less than 1.0% into the liquid hydrolysate due to the relatively mild condition of LHW treatment which has no catalyst for the reaction.

### 3.3. XOS Composition and Total XOS Content

**Figure 3:**
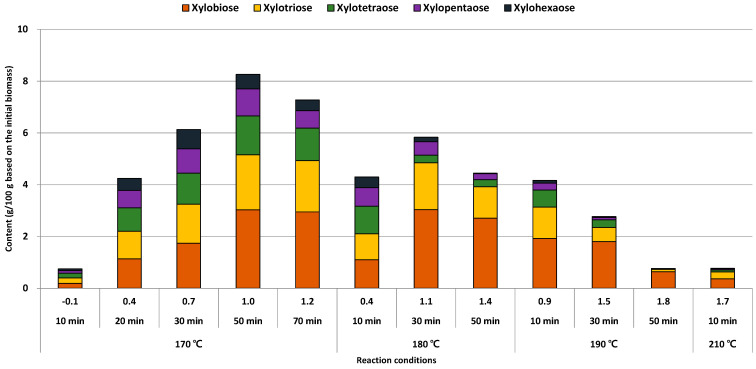
Xylose and XOS content (g/100 g initial biomass) of liquid hydrolysate after LHW treatment from *E. pellita*.

The amount of total XOS increased from 0.8% to 8.3% until 50 min of reaction time at 170 °C, but it decreased slightly (7.3%) as the reaction time extended to 70 min (Figure 3). In this reaction temperature, the conditions of short reaction time (10 min) had a poor production of total XOS than that of conditions of long reaction time (30 min or 50 min). A sufficient reaction time might be required for reducing the degree of polymerization (DP) of XOS from xylan or a high molecular weight of XOS. Meanwhile, a decline of the total XOS content in 70 min condition might be considered that long reaction time excessively reduced the DP of XOS or convert to other product even although the entire xylan was already left from *E. pellita*.

This trend of the total XOS content at 170 °C conditions was changed at 180 °C conditions. As reaction time extended from 30 min to 50 min, total XOS content decreased from 5.8% to 4.5%. A declining trend of total XOS content continued steadily at 190 °C as an increase of reaction time, and the total XOS content was observed as 0.8% at 210 °C for 10 min. Therefore, the extension of reaction time above 180 °C conditions, except for 10 min, maybe not suitable for the production of XOS, because furfural, a derivative from xylose and XOS, was produced vigorously above 180 °C conditions (Figure 2).

Although a single product of the xylose and XOS was not dominantly produced in a specific LHW treatment condition, the ratio of XOS content as the difference of DP changed in this study. Xylobiose was the most produced among the XOS even including xylose, and the maximum amount of xylobiose (3.0%) was obtained at 180 °C for 30 min. In this condition, xylobiose was more detected than xylose (1.8%) because more high reaction temperature with a long reaction time may be required for converting xylose or furfural [28]. The condition of 170 °C for 50 min presented the maximum amount of xylotriose (2.1%), xylotetraose (1.5%), and xylopentaose (1.0%), respectively. Meanwhile, the maximum content of xylohexaose (0.8%) was observed at 170 °C for 30 min. Considering the maximum xylose content (2.2%) at 180 °C for 50 min, the reaction condition was milder as the maximum content of high DP of XOS increased. Thus, the high molecular weight XOS might difficult to keep their chain length even if acid hydrolysis by LHW treatment was performed under mild conditions [29].

The conversion ratio of XOS (Equation (2)) from xylan in the raw material is summarized in Table 3. Generally, the total conversion ratio of XOS was increased/or decreased simultaneously by each DP of XOS, and the variation of the conversion ratio of XOS was roughly constant under all conditions. The maximum conversion ratio of XOS (67.2%) was obtained under 170 °C for 50 min condition, and the maximum conversion ratio of XOS from xylobiose to xylopentaose could be observed under this condition except for xylohexaose. In the case of 170 °C conditions, the conversion ratio of xylohexaose was the most sensitively responded to the extension of the reaction time. Consequently, high DP XOS such as xylohexaose might be difficult to survive even with a slight extension of reaction time. 

### 3.4. Relationship between Combined Severity Factor and Conversion Ratio of XOS

**Figure 4:**
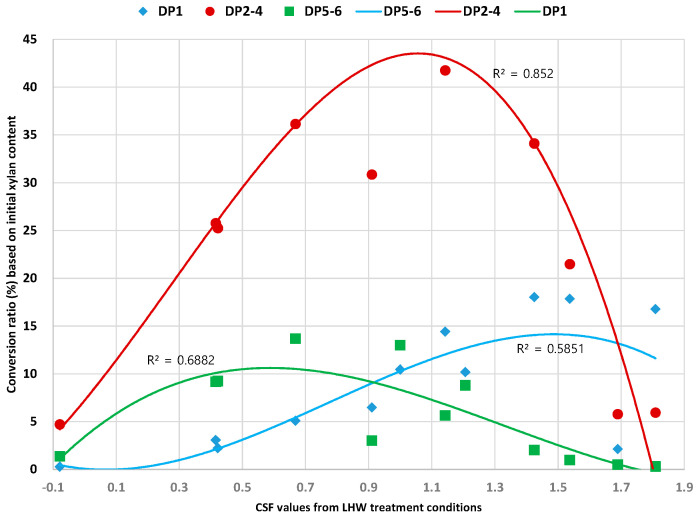
Conversion ratio (%) of xylose and XOS as a DP variation depending on changes of CSF value of LHW treatment conditions.

The conversion ratio of xylose and XOS in the liquid hydrolysate was calculated from xylan content in the raw material (Equation (2)) and is plotted with the values of combined severity factor (CSF) in Figure 4. CSF was invented for quantifying the severity of the treatment conditions, and it has been typically used for evaluating the relationship of conditions and desired product after biomass treatment (Equation (1)) [15]. The CSF values of each condition in this study are stated in Table 1. The conversion ratio was presented with separation of three categories depending on DP; xylose (DP 1) or xylobiose, xylotriose, and xylotetraose (DP 2–4) or xylopentaose and xylohexaose (DP 5–6).

The conversion ratio of DP 2–4 showed the highest value on the ranges from 1.0 to 1.2 CSF value, and it was mostly higher than that of xylose or DP 5–6 on the trend line. The XOS of DP 2–4 has been known as high value molecules for prebiotics application among XOS because these XOS can be readily used by beneficial intestinal microorganisms [30]. Meanwhile, the previous studies reported that when the DP is 5 or more, the availability of microorganisms as prebiotics decreases [2]. Therefore, the DP 2–4 of XOS could be dominantly produced by LHW treatment in the optimal point of CSF values from 1.0 to 1.2. 

The highest conversion ratio of DP 5–6 was determined in the conditions of the low CSF value area, while the xylose conversion ratio was maximized in the condition of the high CSF value area. The DP of XOS decreased by the harsh condition of LHW treatment that was mentioned in the previous section. Besides the R-squared value of the trend line in DP 2-4 was 0.8520 that had a relatively high significance but, that of trend lignin in xylose and DP 5-6 were 0.6882 and 0.5851, respectively. These low significance against CSF value might be induced by a quite difference of influence on biomass among the LHW treatment factors such as reaction temperature, time, and pH in spite of the condition having the same CSF value.

### 3.5. XOS Production Parameters for Process Optimization

Production cost has been considered as the most important factor for all kinds of the product even XOS from biomass because it determines the success of biomass utilization in the industrial level. In this term, we suggested some of the parameters including xylan extraction ratio, XOS purity, low DP XOS ratio, and XOS production score that could evaluate the XOS production cost in Table 4. 

The xylan extraction ratio (Equation (3)) means a proportion of total content of xylose and XOS based on the xylan content in the initial biomass that can represent the xylan yield by LHW treatment. The highest xylan extraction ratio (77.6%) was observed under 170 °C for 50 min, and this condition showed the highest conversion ratio of XOS (67.2%) in Table 3. The xylan extraction ratio in this study might be remarkable by comparing with previous researches that reported 69.1% from miscanthus [31] and 72% from Brewery’s spent grain [32]. 

The second parameter for XOS production is XOS purity (Equation (4)) that is presented the XOS content (DP 2–6) based on the all sugar content in the liquid hydrolysate. Typical impurities for XOS production have been known as sugar derivative products such as furfural, 5-HMF, and formic acid, but other sugars from biomass could reduce the XOS purity. Because the sugar derivative products might be removed by the conventional separation techniques, while the other sugars have similar molecular weight and chemical properties with XOS that makes it hard to separate from XOS. In this study, the XOS purity was determined about 77% under 170 °C conditions that have similar levels to previous studies using LHW treatment: 64.8% from rice husks and 77.6% from corn cob [33].

The third parameter for evaluating the XOS production is the low DP XOS ratio (Equation (5)) which means a proportion of DP 2–4 to DP 2–6 content. According to previous researches, low DP XOS (DP 2–4) presented good physiological performances than that of high DP XOS (above DP 5) [34]. In this term, the low DP ratio in XOS relates to the quality of XOS and XOS can be sold at a high market price by increasing the low DP ratio [35].

**Figure 5:**
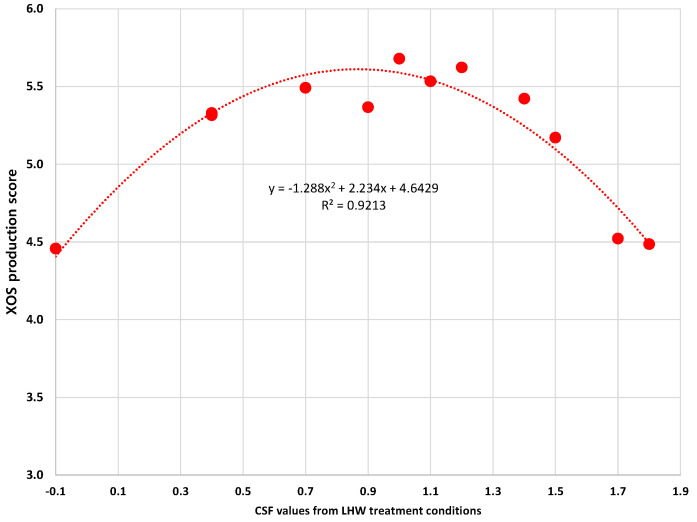
The relationship between XOS production score and CSF values.

The three parameters above mentioned are very critical factors for XOS production because they are deeply related to the production cost and the quality of XOS. However, the previous three parameters alone seem to make it difficult to easily determine which LHW condition is appropriate to produce XOS. For instance, the 190 °C conditions presented high levels of low DP XOS ratios as over 91%, while the xylan extraction ratio and the XOS purity were lower than the results of 170 °C conditions. Thus, we would like to suggest the XOS production score (Equation (6)) which was established by combining the three parameters if they have an equal effect on XOS production. In this study, 170 °C for 50 min condition showed the best XOS production score (5.7). Additionally, we suggest a relationship between the XOS production score and CSF values of LHW conditions (Figure 5). Surprisingly, the relationship showed a high R-squared value (0.9213) and the trend line indicated optimum LHW conditions for XOS production. Although each of the three parameters might not have the same influence on XOS production cost, the XOS produce score could be advanced by considering additional factors such as technical-economic analysis.

### 3.6. Gluccan Conversion by Enzymatic Hydrolysis Using Solid Fractions after LHW Treatment

**Figure 6:**
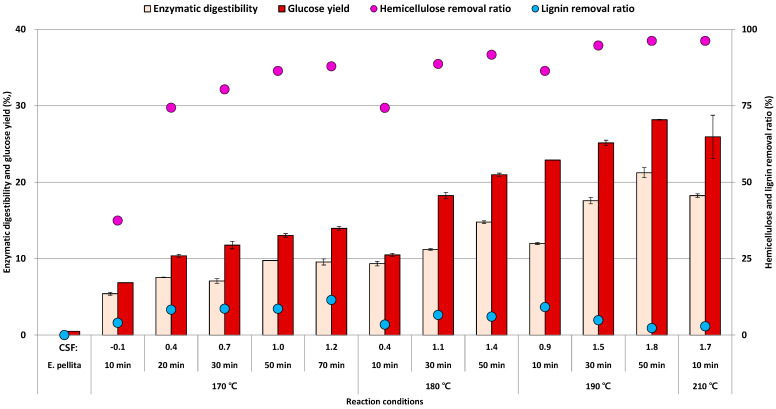
Enzymatic digestibility (%, based on a dry weight of substrate) and glucose yield (%, based on the glucose content in the initial biomass) from *E. pellita* and solid fractions after LHW treatment with hemicellulose and lignin removal ratio (%, based on the content in the initial biomass).

After the enzymatic hydrolysis using solid residues, enzymatic digestibility (Equation (7)) and glucose yield (Equation (8)) were different at the same reaction condition, and glucose yield slightly higher than enzymatic digestibility in most conditions (Figure 6). However, the trend of enzymatic digestibility and glucose yield was similar in terms of the change of LHW treatment conditions. As an increase of the severity factor, the enzymatic digestibility and glucose yield was inclined gradually until 190 °C for 10 min as 21.3% and 28.2%, respectively. However, the enzymatic digestibility and glucose yield was decreased slightly at 210 °C for 10 min as 18.3% and 25.9%, respectively, even though the glucan content in the solid residue did not decrease significantly compared with that of *E. pellita* (Table 2). It is assumed that a pore size in the cell wall was reduced by the dehydration reaction during hydrothermal treatment under the high reaction temperature condition [36]. Besides the degradation products from hemicellulose such as humin might be adhered to the lignin fraction of the LHW treated solid residues, then the complex of humin and lignin reduced cellulase accessibility on the surface of cellulose fibrils [37]. 

The hemicellulose removal ratio was estimated based on the amount of total hemicellulose including xylose, galactose, mannose, and arabinose, and it might be considered to have a certain relationship with the results of enzymatic hydrolysis. Because the enzymatic digestibility and glucose yield were increased as an increase of the hemicellulose removal ratio that revealed at 170 °C for 20–70 min and 180 °C for 10–50 min conditions. Hemicellulose has been believed an inhibitor that served as a barrier concerning cellulase accessibility during enzymatic hydrolysis [38]. Furthermore, hemicellulose removal through hydrothermal treatment can ensure a sufficient space where hemicellulose was in that improves enzyme activity in the cell-wall structure [39]. However, the enzymatic digestibility and glucose yield was increased slightly at 170 °C for 10–20 min conditions in spite of a sharp increase of the hemicellulose removal ratio. Besides the enzymatic hydrolysis and glucose yield were increased under 190 °C conditions than those of 190 °C conditions even though the both of hemicellulose removal ratio under 180 °C and 190 °C conditions were similar. During the LHW treatment, an increase of reaction temperature could contribute to enlargement to the specific surface area that reported in a previous study [40]. On the contrary to the previous study, the hemicellulose removal ratio cannot be considered as a major factor for enzymatic hydrolysis from *E. pellita*. Consequently, the recalcitrance of *E. pellita* slightly reduced by LHW treatment but an additional treatment might be required for achieving high glucose yield.

## 4. Conclusions

In this study, one of the tropical hardwoods, *E. pellita*, was employed for XOS and glucose production by LHW treatment and the relationship between the conditions of severity and the DP of XOS was evaluated. The xylan in *E. pellita* was successfully converted to XOS (max. 67.2%), and the extension of reaction time ensured high XOS production than increasing the reaction temperature. The DP 2–4, which preferred for prebiotics, was dominantly produced than other XOS. XOS production score (max. 5.7 at 170 °C for 50 min condition) was determined by three parameters, xylan extraction ratio, XOS purity, and low DP XOS ratio, that indicated the optimum LHW treatment conditions for producing XOS with the high market price. Meanwhile, moderate glucose yield indicated that the *E. pellita* did not sufficiently decompose for biological treatment through LHW treatment alone.

## Figures and Tables

**Table 1:** Water-insoluble solid (WIS) recovery rate and lignin contents of the *Eucalyptus pellita* and the solid fractions obtained by liquid hot water (LHW) treatment.

Conditions			WIS Recovery Rate (%)		Lignin (%) ^2^	
Reaction Temp. (°C)	Reaction Time (min)	CSF ^1^		AIL ^3^	ASL ^4^	Total ^5^
*E. pellita*			100	32.5 ± 1.0	2.3 ± 0.3	34.8
170	10	−0.1	90.6 ± 0.4	32.3 ± 0.5	1.1 ± 0.1	33.4
	20	0.4	84.7 ± 1.2	30.8 ± 0.3	1.1 ± 0.2	31.9
	30	0.7	82.1 ± 2.1	30.5 ± 1.3	1.3 ± 0.1	31.8
	50	1.0	80.3 ± 0.4	30.9 ± 0.2	0.9 ± 0.0	31.8
	70	1.2	80.8 ± 1.5	29.8 ± 0.2	1.0 ± 0.0	30.8
180	10	0.4	84.1 ± 0.6	32.7 ± 0.7	0.9 ± 0.0	33.6
	30	1.1	78.9 ± 1.2	31.6 ± 0.4	0.9 ± 0.1	32.5
	50	1.4	78.9 ± 0.2	31.3 ± 0.6	1.4 ± 0.1	32.7
190	10	0.9	79.7 ± 1.1	30.5 ± 0.9	1.1 ± 0.1	31.6
	30	1.5	77.3 ± 0.2	32.2 ± 0.1	0.9 ± 0.1	33.1
	50	1.8	76.4 ± 2.3	33.1 ± 0.2	0.9 ± 0.0	34.0
210	10	1.7	76.6 ± 1.5	32.7 ± 1.4	1.1 ± 0.2	33.8

^1^ Combined severity factor; ^2^ Based on dry weight of biomass; ^3^ Acid-insoluble lignin; ^4^ Acid-soluble lignin; ^5^ Sum of AIL and ASL content.

**Table 2:** Structural sugar contents of *E. pellita* and the solid fractions obtained by LHW treatment.

Conditions		Structural Sugars (%) ^1^					
Reaction Temp. (°C)	Reaction Time (min)	Glucan	Xylan	Galactan	Mannan	Arabinan	Total ^2^
*E. pellita*		50.9 ± 1.5	10.8 ± 0.4	1.6 ± 0.1	0.6 ± 0.0	0.3 ± 0.0	64.2
170	10	47.5 ± 0.5	7.7 ± 0.5	0.6 ± 0.1	ND ^3^	ND	55.8
	20	46.5 ± 0.7	3.1 ± 0.2	0.3 ± 0.1	ND	ND	49.9
	30	46.3 ± 2.7	2.6 ± 0.3	ND	ND	ND	48.9
	50	47.4 ± 2.3	1.8 ± 0.3	ND	ND	ND	49.2
	70	46.7 ± 0.6	1.6 ± 0.2	ND	ND	ND	48.3
180	10	46.3 ± 1.5	3.4 ± 0.3	ND	ND	ND	49.7
	30	45.4 ± 0.0	1.5 ± 0.1	ND	ND	ND	46.9
	50	43.4 ± 0.3	1.1 ± 0.3	ND	ND	ND	44.5
190	10	46.4 ± 0.9	1.8 ± 0.4	ND	ND	ND	48.2
	30	42.7 ± 2.3	0.7 ± 0.0	ND	ND	ND	43.4
	50	45.2 ± 2.0	0.5 ± 0.1	ND	ND	ND	45.7
210	10	44.6 ± 1.3	0.5 ± 0.1	ND	ND	ND	45.1

^1^ Based on dry weight of biomass; ^2^ Sum of structural sugar content; ^3^ Not detected.

**Table 3:** Conversion ratio (%) of XOS in the liquid hydrolysate after LHW treatment.

Conditions		Conversion Ratio of XOS (%) ^1^					
Reaction Temp. (°C)	Reaction Time (min)	Xylobiose	Xylotriose	Xylotetraose	Xylopentaose	Xylohexaose	Total ^2^
170	10	1.5	1.8	1.5	0.8	0.5	6.1
	20	9.2	8.7	7.4	5.5	3.7	34.5
	30	14.1	12.3	9.7	7.6	6.1	49.9
	50	24.7	17.2	12.2	8.5	4.5	67.2
	70	24.0	16.1	10.2	5.5	3.3	59.1
180	10	9.0	8.2	8.6	5.8	3.4	35.0
	30	24.7	14.7	2.4	4.3	1.4	47.4
	50	22.0	9.9	2.2	1.9	0.2	36.1
190	10	15.6	9.9	5.4	2.2	0.8	33.9
	30	14.6	4.5	2.4	0.7	0.3	22.5
	50	5.2	0.7	0.0	0.0	0.3	6.3
210	10	2.9	2.2	0.6	0.3	0.2	6.3

^1^ Based on the initial xylan content; ^2^ Sum of conversion ratio.

**Table 4:** Xylan extraction ratio, XOS purity, low DP XOS ratio (%), and XOS production score with LHW treatment conditions.

Conditions			Xylan Extraction Ratio (%)	XOS Purity (%)	Low DP XOS Ratio (%)	XOS Production Score
Reaction Temp. (°C)	Reaction Time (min)	CSF				
170	10	−0.1	6.4	57.8	77.4	4.5
	20	0.4	36.7	77.0	73.2	5.3
	30	0.7	55.0	77.8	72.5	5.5
	50	1.0	77.6	76.5	80.6	5.7
	70	1.2	69.3	71.2	85.1	5.6
180	10	0.4	38.1	76.2	73.7	5.3
	30	1.1	61.9	62.8	88.1	5.5
	50	1.4	54.2	5.18	94.4	5.4
190	10	0.9	40.4	63.5	91.1	5.4
	30	1.5	40.4	38.5	95.5	5.2
	50	1.8	23.1	14.0	95.1	4.5
210	10	1.7	8.4	42.9	91.8	4.5

## Data Availability

Data is contained within the article.

## References

[1] Aachary A.A., Prapulla S.G. (2011). Xylooligosaccharides (XOS) as an emerging prebiotic: Microbial synthesis, utilization, structural characterization, bioactive properties, and applications. Compr. Rev. Food. Sci. Food Saf..

[2] Gullόn P., Moura P., Esteves M.a.P., Girio F.M., Domínguez H., Parajό J.C. (2008). Assessment on the fermentability of xylooligosaccharides from rice husks by probiotic bacteria. J. Agric. Food. Chem..

[3] Grootaert C., Delcour J.A., Courtin C.M., Broekaert W.F., Verstraete W., Van de Wiele T. (2007). Microbial metabolism and prebiotic potency of arabinoxylan oligosaccharides in the human intestine. Trends Food Sci. Technol..

[4] Taniguchi H. (2004). Carbohydrate research and industry in Japan and the Japanese Society of Applied Glycoscience. Starke.

[5] Chen M.-H., Bowman M.J., Cotta M.A., Dien B.S., Iten L.B., Whitehead T.R., Rausch K.D., Tumbleson M., Singh V. (2016). Miscanthus× giganteus xylooligosaccharides: Purification and fermentation. Carbohydr. Polym..

[6] Brienzo M., Carvalho W., Milagres A.M. (2010). Xylooligosaccharides production from alkali-pretreated sugarcane bagasse using xylanases from Thermoascus aurantiacus. Appl. Biochem. Biotechnol..

[7] Otieno D.O., Ahring B.K. (2012). A thermochemical pretreatment process to produce xylooligosaccharides (XOS), arabinooligosaccharides (AOS) and mannooligosaccharides (MOS) from lignocellulosic biomasses. Bioresour. Technol..

[8] Akpinar O., Erdogan K., Bostanci S. (2009). Production of xylooligosaccharides by controlled acid hydrolysis of lignocellulosic materials. Carbohydr. Res..

[9] Surek E., Buyukkileci A.O. (2017). Production of xylooligosaccharides by autohydrolysis of hazelnut (Corylus avellana L.) shell. Carbohydr. Polym..

[10] Florence R.G. (2004). Ecology and Silviculture of Eucalypt Forests.

[11] Yu Q., Zhuang X., Yuan Z., Wang Q., Qi W., Wang W., Zhang Y., Xu J., Xu H. (2010). Two-step liquid hot water pretreatment of Eucalyptus grandis to enhance sugar recovery and enzymatic digestibility of cellulose. Bioresour. Technol..

[12] Hung T.D., Brawner J.T., Meder R., Lee D.J., Southerton S., Thinh H.H., Dieters M.J. (2015). Estimates of genetic parameters for growth and wood properties in Eucalyptus pellita F. Muell. to support tree breeding in Vietnam. Ann. For. Sci..

[13] Dombro D.B. (2010). Eucalyptus Pellita: Amazonia Reforestation’s Red Mahogany.

[14] Setiadji R., Husin A.A. (2012). Utilization of Eucalyptus oil refineries waste for cement particle board. IJSCET.

[15] Abatzoglou N., Chornet E., Belkacemi K., Overend R.P. (1992). Phenomenological kinetics of complex systems: The development of a generalized severity parameter and its application to lignocellulosics fractionation. Chem. Eng. Sci..

[16] Sluiter A., Hames B., Ruiz R., Scarlata C., Sluiter J., Templeton D. (2006). Determination of sugars, byproducts, and degradation products in liquid fraction process samples. Gold. Natl. Renew. Energy Lab..

[17] Jang S.-K., Kim J.-H., Jeong H., Choi J.-H., Lee S.-M., Choi I.-G. (2018). Investigation of conditions for dilute acid pretreatment for improving xylose solubilization and glucose production by supercritical water hydrolysis from Quercus mongolica. Renew. Energy.

[18] Zhuang X., Wang W., Yu Q., Qi W., Wang Q., Tan X., Zhou G., Yuan Z. (2016). Liquid hot water pretreatment of lignocellulosic biomass for bioethanol production accompanying with high valuable products. Bioresour. Technol..

[19] Sun X.-F., Sun R., Fowler P., Baird M.S. (2005). Extraction and characterization of original lignin and hemicelluloses from wheat straw. J. Agric. Food. Chem..

[20] Cara C., Romero I., Oliva J.M., Sáez F., Castro E. (2007). Liquid hot water pretreatment of olive tree pruning residues. Applied Biochemistry and Biotechnology.

[21] Sjostrom E. (1993). Wood Chemistry: Fundamentals and Applications.

[22] Hendriks A., Zeeman G. (2009). Pretreatments to enhance the digestibility of lignocellulosic biomass. Bioresour. Technol..

[23] Kumar R., Singh S., Singh O.V. (2008). Bioconversion of lignocellulosic biomass: Biochemical and molecular perspectives. J. Ind. Microbiol..

[24] Lü H., Shi X., Li Y., Meng F., Liu S., Yan L. (2017). Multi-objective regulation in autohydrolysis process of corn stover by liquid hot water pretreatment. Chin. J. Chem. Eng..

[25] Feather M.S., Harris D.W., Nichols S.B. (1972). Routes of conversion of D-xylose, hexuronic acids, and L-ascorbic acid to 2-furaldehyde. J. Org. Chem.

[26] Morone A., Apte M., Pandey R. (2015). Levulinic acid production from renewable waste resources: Bottlenecks, potential remedies, advancements and applications. Renew. Sustain. Energy Rev..

[27] Girisuta B., Janssen L., Heeres H. (2006). A kinetic study on the decomposition of 5-hydroxymethylfurfural into levulinic acid. Green Chem..

[28] Garrote G., Domínguez H., Parajó J.C. (1999). Mild autohydrolysis: An environmentally friendly technology for xylooligosaccharide production from wood. J. Chem. Technol. Biotechnol..

[29] Parajó J., Garrote G., Cruz J., Dominguez H. (2004). Production of xylooligosaccharides by autohydrolysis of lignocellulosic materials. Trends Food Sci. Technol..

[30] Huang C., Lai C., Wu X., Huang Y., He J., Huang C., Li X., Yong Q. (2017). An integrated process to produce bio-ethanol and xylooligosaccharides rich in xylobiose and xylotriose from high ash content waste wheat straw. Bioresour. Technol..

[31] Chen M.-H., Bowman M.J., Dien B.S., Rausch K.D., Tumbleson M., Singh V. (2014). Autohydrolysis of Miscanthus x giganteus for the production of xylooligosaccharides (XOS): Kinetics, characterization and recovery. Bioresour. Technol..

[32] Carvalheiro F., Esteves M., Parajó J., Pereira H., Gırio F. (2004). Production of oligosaccharides by autohydrolysis of brewery’s spent grain. Bioresour. Technol..

[33] Garrote G., Falqué E., Domínguez H., Parajó J.C. (2007). Autohydrolysis of agricultural residues: Study of reaction byproducts. Bioresour. Technol..

[34] Ho A.L., Kosik O., Lovegrove A., Charalampopoulos D., Rastall R.A. (2018). In vitro fermentability of xylo-oligosaccharide and xylo-polysaccharide fractions with different molecular weights by human faecal bacteria. Carbohydr. Polym..

[35] Ruiz E., Gullón B., Moura P., Carvalheiro F., Eibes G., Cara C., Castro E. (2017). Bifidobacterial growth stimulation by oligosaccharides generated from olive tree pruning biomass. Carbohydr. Polym..

[36] Ertas M., Han Q., Jameel H., Chang H.-m. (2014). Enzymatic hydrolysis of autohydrolyzed wheat straw followed by refining to produce fermentable sugars. Bioresour. Technol..

[37] Filiciotto L., Balu A.M., Van der Waal J.C., Luque R. (2018). Catalytic insights into the production of biomass-derived side products methyl levulinate, furfural and humins. Catal. Today.

[38] Mussatto S.I., Fernandes M., Milagres A.M., Roberto I.C. (2008). Effect of hemicellulose and lignin on enzymatic hydrolysis of cellulose from brewer’s spent grain. Enzyme Microb. Technol..

[39] Yoshida M., Liu Y., Uchida S., Kawarada K., Ukagami Y., Ichinose H., Kaneko S., Fukuda K. (2008). Effects of cellulose crystallinity, hemicellulose, and lignin on the enzymatic hydrolysis of Miscanthus sinensis to monosaccharides. Biosci. Biotechnol. Biochem..

[40] Thompson D.N., Chen H.-C., Grethlein H.E. (1992). Comparison of pretreatment methods on the basis of available surface area. Bioresour. Technol..

